# Using expert knowledge and modeling to define mangrove composition, functioning, and threats and estimate time frame for recovery

**DOI:** 10.1002/ece3.1085

**Published:** 2014-05-08

**Authors:** Nibedita Mukherjee, William J Sutherland, Md Nabiul I Khan, Uta Berger, Nele Schmitz, Farid Dahdouh-Guebas, Nico Koedam

**Affiliations:** 1 Laboratory of Systems Ecology and Resource Management, Université Libre de BruxellesCP 169, Avenue F.D. Roosevelt 50, B-1050, Brussels, Belgium; 2 Laboratory of Plant Biology and Nature Management, Vrije Universiteit BrusselPleinlaan 2, B-1050, Brussels, Belgium; 3 Conservation Science Group, Department of Zoology, University of CambridgeCB2 3EJ, Cambridge, UK; 4 Institute of Forest Growth and Forest Computer SciencesTU Dresden, P.O. 1117, 01735, Tharandt, Germany; 5 Institute of Botany, BOKU ViennaGregor Mendel Str. 33, 1180, Vienna, Austria

**Keywords:** Coastal development, Delphi technique, ecosystem functioning, global mangroves, individual-based modeling, policy

## Abstract

Mangroves are threatened worldwide, and their loss or degradation could impact functioning of the ecosystem. Our aim was to investigate three aspects of mangroves at a global scale: (1) their constituents (2) their indispensable ecological functions, and (3) the maintenance of their constituents and functions in degraded mangroves. We focused on answering two questions: “What is a mangrove ecosystem” and “How vulnerable are mangrove ecosystems to different impacts”? We invited 106 mangrove experts globally to participate in a survey based on the Delphi technique and provide inputs on the three aspects. The outputs from the Delphi technique for the third aspect, *i.e*. maintenance of constituents and functions were incorporated in a modeling approach to simulate the time frame for recovery. Presented here for the first time are the consensus definition of the mangrove ecosystem and the list of mangrove plant species. In this study, experts considered even monospecific (tree) stands to be a mangrove ecosystem as long as there was adequate tidal exchange, propagule dispersal, and faunal interactions. We provide a ranking of the important ecological functions, faunal groups, and impacts on mangroves. Degradation due to development was identified as having the largest impact on mangroves globally in terms of spatial scale, intensity, and time needed for restoration. The results indicate that mangroves are ecologically unique even though they may be species poor (from the vegetation perspective). The consensus list of mangrove species and the ranking of the mangrove ecological functions could be a useful tool for restoration and management of mangroves. While there is ample literature on the destruction of mangroves due to aquaculture in the past decade, this study clearly shows that more attention must go to avoiding and mitigating mangrove loss due to coastal development (such as building of roads, ports, or harbors).

## Introduction

Mangrove ecosystems are predominantly tropical and subtropical (30°N to 37°S) tidally influenced coastal wetlands, present in 123 countries (Feller et al. 2010; Spalding et al. 2010). Mangroves are estimated to be the most carbon-rich forests in the tropics per unit area containing approximately 1023 Mg carbon·ha^−1^ (Donato et al. 2011). In spite of their ecological and socioeconomic significance, mangrove area has declined by 30–50% in the past 50 years, a higher rate than most other biomes (Balmford et al. 2002). The loss and degradation of biodiversity in mangrove ecosystems could impair their ecosystem functioning and they may functionally disappear from the earth before the next century (Duke et al. 2007). Ecosystem functioning has been defined by Reiss et al. (2009) as the combination of three ecosystem characteristics: (1) ecosystem attributes and properties, (2) ecosystem processes, and (3) maintenance of ecosystem processes and properties over time (and we follow this definition throughout the text). Change in the biodiversity and ecosystem functioning in mangroves may directly or indirectly impact millions of people dependent on mangroves for ecosystem services (e.g., coastal protection) and livelihoods (Walters et al. 2008).

In biodiversity and ecosystem functioning research, mangroves and other aquatic ecosystems have received less attention than terrestrial ecosystems, such as grasslands or drylands, which have been the focus of most research (73%) (Caliman et al. 2009). Moreover, currently there is substantial evidence from other ecosystems that the loss of biodiversity has a considerable impact on ecosystem functioning and consequently on the survival of humans and other organisms (Petchey et al. 2004; Zavaleta and Hulvey 2004; Hector and Bagchi 2007; Loreau 2010; Cardinale et al. 2012). In addition, Barbier (2011) has stated that investigating ecosystem functioning may be particularly important for those ecosystems that both provide a disproportionately large contribution toward ecosystem services and are highly threatened, for example coastal ecosystems. Hence, ecosystem functioning in mangroves deserves urgent research attention, as mangrove forests are both socioeconomically important and highly threatened.

To develop a better understanding of ecosystem functioning in mangroves, it is important to answer two key questions: “What is a ‘mangrove ecosystem’?” and “How vulnerable are mangrove ecosystems to different impacts?” These overarching questions are best answered from a global perspective rather than a local one because mangroves cover a wide biogeographical range (spanning 65° of latitude) and are remarkably similar in their floristic composition. Mangrove plants comprise only about 70 plant species worldwide with three genera (*Acrostichum, Avicennia,* and *Rhizophora*) having a global distribution (Spalding et al. 2010). As mangrove ecosystems are impacted throughout their range, a global synthesis is the only approach that will enable the identification of the key impacts and the subsequent prioritization of conservation efforts for functional recovery.

Until now, these questions have not been answered satisfactorily from a global perspective because of three main challenges: (1) *Data gaps*: One of the largest bottlenecks is the lack of global datasets on mangroves. For instance, international databases such as GlopNet or TRY have little trait information for mangrove plants when compared to other forest plant species (Wright et al. 2002; Kattge et al. 2011). (2) *Complexity*: Mangrove ecosystems vary considerably in their attributes (structure, function, and anthropogenic pressures) in different parts of the world. These range from Southeast Asian muddy substrates on deltaic settings having over thirty mangrove species to Caribbean Island sandy substrates having two to four mangrove species; furthermore, the gradients in environmental parameters such as salinity, grain size, and tidal range can be considerable (Duke 1992; Feller et al. 2010). Given this diversity, it can be difficult to prioritize the key functional attributes likely to be critical for restoring biodiversity and ecosystem functioning in degraded mangrove forests. In addition, anthropogenic pressures can be expected to affect mangrove ecology and influence the time required for regaining functionality in degraded mangroves. (3) *Lack of consensus*: As early as 1979, Snedaker, an expert on mangroves, considered “mangroves” a scientifically ambiguous term, as it is used for both the plants and the whole ecosystem (Snedaker 1979). Even today, there is little consensus on what constitutes “mangrove species” because expert opinion on which plant species can be considered to be mangroves still differs considerably. For instance, the recent World Atlas of Mangroves (Spalding et al. 2010), which mapped the global distribution of mangroves, had a different plant species list than the analysis of global mangrove threats and species vulnerability (Polidoro et al. 2010). The mangrove literature is rife with (often ambiguous) terminology such as “mangrove associates”, “true mangrove”, “major mangrove”, “minor mangrove” *sensu* Tomlinson (1986). This ambiguity in the ecological classification of mangroves has serious implications for research, management, and restoration of mangroves (Jayatissa et al. 2002; Veenakumari and Prashanth 2009). Similar ambiguity exists in delimiting mangrove faunal associations over wide geographic scales (Sheaves 2012).

Given the complexity of the issues, it is difficult for one person or a few individuals to arrive at a comprehensive view on mangroves as an ecosystem based purely on their own observations. Consolidating the expertise from a wide range of experts, each of whom have more than 10,000 h (amounting to greater than 10 years) of focused engagement with the subject (Ericsson et al. 2006), would be an important first step in addressing ecosystem functioning and functional recovery in mangrove ecosystems (Sutherland et al. 2011; Sutherland 2013). Moreover, consensus from a wide range of experts would be the only solution to arrive at a working definition for the mangrove ecosystem and to resolve the lack of consensus in mangrove species listing. The expert-based iterative Delphi technique (see Methods section) is relevant in this context as it has been used for generating consensus and filling in data gaps in ecology and is already known to be highly structured and rigorous (MacMillan and Marshall 2006; O'Neill et al. 2008; Swor and Canter 2011; Martin et al. 2012).

In addition to consensus on complex issues, quantifiable results (e.g., through modeling) may also be vital for conservation decision making. Thus, a combination of expert opinion and modeling has been suggested to be ideal by Sutherland (2006) and has been used in this study.

In this paper, we aimed at answering the overarching questions about what a mangrove system is and how vulnerable it is to a range of different impacts, by looking into the three aspects of ecosystem functioning following the definition of Reiss et al. (2009). These three aspects were translated into five specific sub-sections, as given below:

### Constituents of the ecosystem

1. What is the consensus definition of a mangrove ecosystem?

All existing definitions for the mangrove ecosystem are heavily biased toward the vegetation (Appendix S1). Our aim was to initiate thought and seek new opinion on a holistic definition of a mangrove ecosystem rather than one based on vegetation alone.

**(2)** What are the constituents of the mangrove ecosystem?

Three aspects were discussed in this phase of the research:

*Which plant species can be considered characteristic of mangrove ecosystems?:* A consolidated species list is essential for identifying species that could be (1) counted while estimating area under mangrove cover or calculating change in species richness, (2) restored or planted in a degraded mangrove, and (3) prioritized while planning conservation efforts. This could only be addressed through expert knowledge.
*Which faunal groups can be commonly observed in a mangrove?:* Although there is a plethora of published studies on crabs and birds (Cannicci et al. 2007; Kristensen 2008; Luther and Greenberg 2011) and some local-scale studies have been conducted on other faunal groups, we know very little about global patterns in mangrove fauna. Hence, we were interested in identifying the faunal groups that are commonly observed in a mangrove forest.
*Is one plant species sufficient for an ecosystem?* A notable characteristic of mangrove forests is their strikingly low floristic diversity in almost all parts of their range, even though faunal diversity may be high (Ellison 2002; Nagelkerken et al. 2008). Given the low plant species diversity, we were interested to know whether a mono-specific (having only one tree species) natural or planted mangrove forest would generally be considered to be a mangrove ecosystem. If not, then what would be needed to have a mangrove ecosystem?

### Ecological functions

**(3)** Which functions are perceived to be indispensable in mangrove ecosystems?

While all functions are important for the overall functioning of the mangrove ecosystem, maintenance of the highly ranked functions could be used as an initial step in estimating the success of the restoration process. We define “indispensable functions” as those ecological properties and processes of the mangrove ecosystem that are essential for the overall functioning in a mangrove ecosystem, for example nitrogen fixation or import and export of carbon. These are also the functions that one would typically aim to restore in a degraded mangrove in a restoration programme.

### Maintenance of constituents and functions

**(4)** What are the spatial scales and intensities of the different impacts to mangroves?

Ten major impacts to mangroves were identified from the literature (Valiela et al. 2001; Duke et al. 2007). The ranking of these ten impacts would generate an indicator of the most pressing impacts on mangroves that is difficult to generate in any other way (i.e., through meta-analysis) because site-specific data at the global scale are lacking.

**(5)** How long will it take to restore ecological functionality in degraded mangroves?

We wished to know whether degraded mangroves can regain functionality either through (1) natural restoration or (2) human-assisted restoration. “Human-assisted” includes any kind of human intervention ranging from restoring hydrology to direct plantation (Lewis and Gilmore 2007). If restoration is possible, then we wished to know the time frame required for the restoration process (under natural or human-assisted scenarios) as a function of the scale and intensity of impacts.

To our knowledge, this is the first expert-community-based definition of mangrove ecosystems, mangrove composition, function, and impacts. It is also the first study that attempts to assess the vulnerability of mangroves to different impacts and predict the time frame for recovery based on both modeling and pooled expertise of mangrove experts.

## Methods

### The Delphi technique

#### Brief description of the method

**Figure 1:**
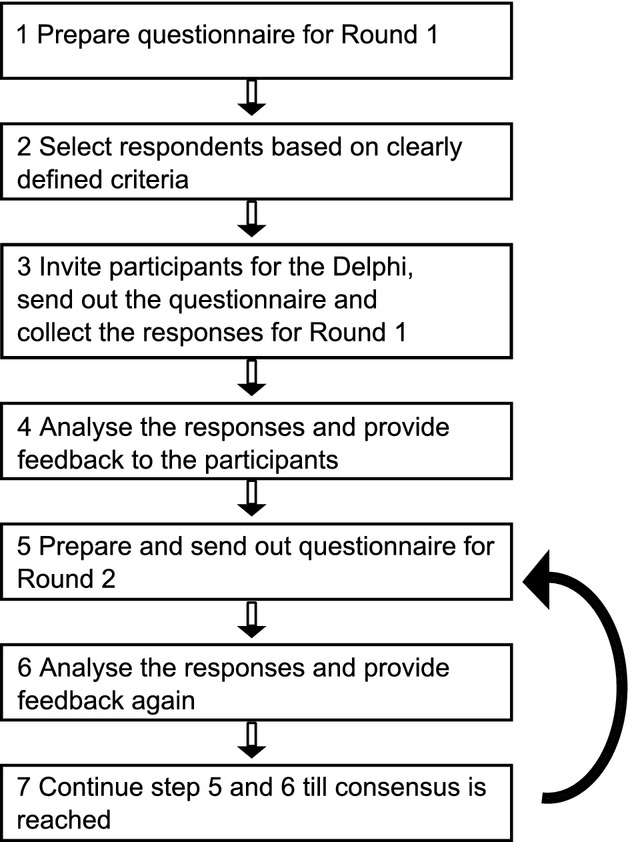
Flowchart representing the Delphi technique aimed at gathering consensus. The number of rounds was limited to two in this study.

The Delphi technique is defined as “a method for structuring a group communication process so that the process is effective in allowing a group of individuals as a whole to deal with a complex problem” (Turoff 1970). The procedure broadly consists of the six steps given below (Fig. 1). Key features of this method are: (1) structuring of information flow, (2) feedback to participants, and (3) anonymity of participants. The underlying concept is that the entire group of participants can evaluate the information produced by the group and weigh dissenting views; the consensus increases from round to round (Steyaert and Lisoir 2005). Individual participants may reconsider or explain their suggestions based upon their evaluation of new information provided. Essentially, the Delphi technique transforms diverse individual knowledge to create a collective wisdom without the domination of individual views (Dalkey and Helmer 1963; De Villiers et al. 2005).

#### Mangrove Delphi

In this study, experts were asked to provide inputs on three following aspects of biodiversity and ecosystem functioning in mangroves: (1) constituents, (2) functions, and (3) maintenance of constituents and functions. We invited the selected 106 mangrove experts (scientists, managers, and on-ground conservationists) to participate in the survey (see Appendix S1 for criteria of selection of experts). The Delphi technique consisted of two rounds, conducted within a time frame of 4 months (November 5, 2011 to March 5, 2012). In the first round, eleven questions were asked. In the second round, the questionnaire was slightly modified based on the comments received in the first round (typical for the Delphi technique), and eighteen questions were asked. Unless otherwise stated, the results from the second round of the Delphi technique are presented here. In some questions, experts were asked to rank options on a Likert scale of 1–5 (Likert 1932). In the case of questions with multiple options involving ranking, the Likert scale scores for each option were given corresponding weights (e.g., Likert score 1 = weight 1, Likert score 5 = weight 5) and multiplied by the number of votes for that option. Later, these weighted scores were converted to a percentage scale.

### Constituents of the ecosystem

#### Consensus definition

Experts were provided with eight definitions of mangrove ecosystems drawn from the published literature and were given the option to suggest their own (see Appendix S1). In the second round, experts were asked to choose the definition that they preferred the most.

#### Mangrove plant species list

Experts were asked to select the species they considered a mangrove plant, based on an updated list of mangrove plants obtained from a global database (Alemán et al. 2010) following the APG III systematics (Stevens 2001 and onwards). Care was taken to ensure that the local species lists would be covered in the global list. This question was asked only in the first round as we obtained consensus in the first round itself. Species with more than 50% votes were included in the consensus list.

#### Faunal groups

Experts were asked to rank the occurrence of ten faunal groups in mangroves adapted from a comprehensive review on mangrove fauna by Nagelkerken et al. (2008). Rankings were based on direct and indirect observations by the experts or their research teams during any field programmes and regardless of whether the species were the target of their research or not. Observations comprised: (1) direct observations, such as sightings, calls heard, or sample collection; and (2) indirect observations, such as fecal matter, burrows, feathers, scales, or nests.

#### Is one plant species sufficient for an ecosystem?

Experts were asked whether a mono-specific (having only one tree species) natural or planted mangrove forest would generally be considered to be a mangrove ecosystem. If not, then we asked the experts what would be needed to have a mangrove ecosystem.

### Ecological functions

Experts were asked to (1) identify ecological functions that they perceive to be indispensable to a “healthy” mangrove and (2) rank the functions on a Likert scale of 1–5 (Likert 1932). In the first round of the survey, we provided a preliminary list of functions (such as nitrogen fixation, wave attenuation, creation of spatial niche) based on peer-reviewed articles on mangrove ecological functionality (Badola and Hussain 2005; Bosire et al. 2008; Kristensen et al. 2008; Feller et al. 2010; Vovides et al. 2011). In the second round, the list was enlarged to incorporate the input from the first round. It must be noted that this question was not aimed to address the anthropocentric “use values” or ecosystem services of a mangrove. This aspect has not been dealt with in this paper.

### Maintenance of constituents and functions

#### Spatial scale and intensity of impacts

Experts were asked to choose one country where they had carried out the largest share of their fieldwork. For that country, experts were asked to rank ten impacts to mangroves based on the geographic scale and intensity of the impact. These rankings were also used as indicators in the subsequent modeling approach (see below).

#### Time frame for recovery

The experts were asked whether mangroves could regain functionality in the degraded areas either through (1) natural restoration or (2) human-assisted restoration. The experts were asked to refer to the list of ranked ecological functions from the previous section, while considering the possibility of regain of functionality. If restoration was possible, then the respondents were asked to estimate the amount of time required for the restoration process as a function of the scale and intensity of impacts previously identified by them under two scenarios: natural restoration and human-assisted restoration of functionality. The experts were given four options for the time frame of recovery: 0–10 years, 10–20 years, 20–30 years, and greater than 30 years.

The responses were aggregated as short term (i.e., less than 20 years) and long term (i.e., above 20 years). The responses to natural and human-assisted restoration were also categorized in the short-term and long-term time frames. Assuming that developed nations may have different pressures on mangroves than developing nations, these responses were further divided into two categories: highly developed and less developed countries (adapted from the categorization scheme used in the Human Development Index, 2011, UNDP 2013).

#### Prediction of recovery time based on modeling in KiWi

**Figure 2:**
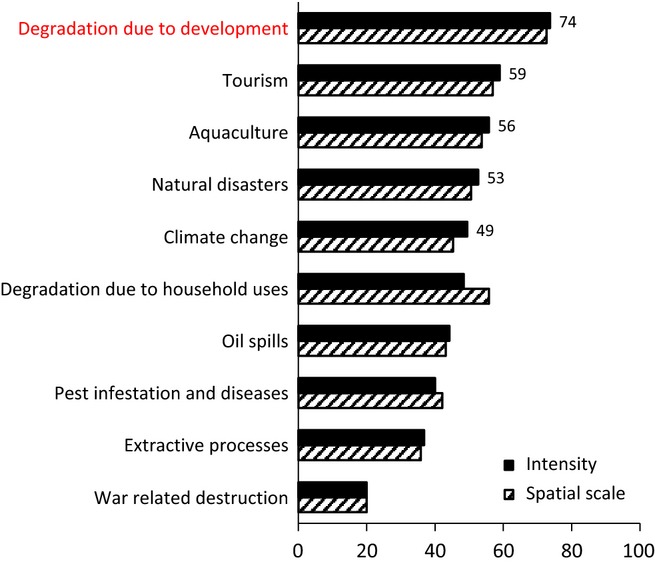
Intensity and spatial scale of ten impacts on mangroves in the study sites of the experts. Experts had selected one country that he/she was most familiar with, for estimating the impacts. Development related degradation (indicated in red) was found to have the greatest impact on mangroves, both in terms of intensity and spatial scale across all study sites.

In addition to the expert-based time frame for functional recovery, we used the well-established mangrove forest simulator KiWi (Berger and Hildenbrandt 2000) to predict the vulnerability of mangroves to the ten most common types of disturbances (e.g., degradation due to development and natural disasters) identified by the Delphi technique. The disturbances were implemented as model secenarios using the information obtained from the Delphi technique spatial scale and intensity of impact (Fig. 2). For example, the information on the spatial scale and intensity of an impact was translated into a reduction in tree density and tree growth randomly in the specified area. Regeneration was also reduced (e.g., after soil degradation and oil spills) with recruitment rates modified based on the spatial scale and intensity score given by the experts (Table 1).

**Table 1:** Parameter values describing the impact scenarios used for simulation experiments in the individual-based model (KiWi). All other model parameters are the same as indicated in Fontalvo-Herazo et al. (2011)

No.	Parameters	Parameter values	Source
1	Size	1 ha	
2	Location	VJR Matang	Putz and Chan (1986), Gong and Ong (1995)
3	Biomass	300 *t* ha^−1^	Putz and Chan (1986), Gong and Ong (1995)
4	Species	*Rhizophora apiculata*	
5	Height- max	50 m	Putz and Chan (1986), Gong and Ong (1995)
6	Spatial scale of impact (i)	Spatial scale from Delphi for impact (i)	
7	Seedling recruitment in the unimpacted area	390 ha^−1^	Arnaud et al. (2011)
8	Seedling recruitment in the impacted area	390 × (1−intensity of impact (i) × (1−spatial scale of impact (i) in Delphi))	
9	Tree density in unimpacted area	1344 ha^−1^	Arnaud et al. (2011)
10	Tree density in impacted area	1344 × (1−intensity of impact (i) × (1−spatial scale of impact (i) in Delphi))	
11	Growth in unimpacted area (G)	Normal growth function G	
12	Growth in impacted area	G × (1−intensity of impact (i) × (1−spatial scale of impact (i) in Delphi))	
13	Basal area in unimpacted area	31.89 m^2^·ha^−1^	Arnaud et al. (2011)
14	Basal area in impacted area	31.89 × (1−intensity of impact (i) in Delphi)	
15	Run time	Until asymptote for biomass or basal area achieved	
16	Number of simulations	100	

#### Brief description of the KiWi model

The KiWi model (Berger and Hildenbrandt 2000) is based on the pattern-oriented modeling approach (Grimm et al. 2005; Fontalvo-Herazo et al. 2011) and was originally explored in the context of a neotropical mangrove forest (Piou et al. 2007; Berger et al. 2008). The model has already been tested and shown to adequately reproduce the forest density and size class distribution of a mangrove plantation in Malaysia (Fontalvo-Herazo et al. 2011), and the regeneration of mangrove stands on abandoned rice fields (Berger et al. 2006). It has also been used to assess the potential of small scale canopy disturbances in driving Vietnamese mangrove plantations towards more natural conditions (Vogt et al. 2013) and to investigate desynchronizing effects of lightning strikes on cyclic forest dynamics in mangrove plantations (Kautz et al. 2011). KiWi has also been used to understand the zonation patterns that have emerged in mangrove forests recovering from hurricane Hattie 1961 on Calabash island in Belize (Piou et al. 2006), and for testing the validity of the intermediate disturbance hypothesis in the context of species-poor mangrove systems (Piou et al. 2008).

The KiWi model is spatially explicit and it describes individual trees by their stem variables (position, diameter, and height) and the field of neighborhood, which is defined as the area within which a tree influences and is influenced by its neighbors. The model assumes that the growth of the trees depends on the tree's age, environmental conditions, and neighborhood competition. Natural tree mortality increases with growth reduction and older trees or trees, heavily suppressed by their neighbours, die. However, the growth reduction in every tree was evaluated as a moving average over the last 5 years. Hence, smaller trees might recover from growth reduction as soon as they are released by neighborhood competition induced by the death of one of its neighbours (see Berger and Hildenbrandt (2000) for details). In addition to the natural mortality, trees might die due to external impacts, and this was implemented as death of a certain fraction of trees (see simulation experiments described below).

Further, the model assumed seed dispersal to occur at random. The establishment of new saplings at a particular location depended on both environmental conditions and competition strength exerted by the already established trees (estimated by the sum of their field of neighborhoods, see Berger and Hildenbrandt 2000 for details).

#### Modeling for mangrove impacts

We created a virtual natural forest of one hectare using the empirical (time series) data from Matang mangroves to parameterize the model (Putz and Chan 1986; Ong et al. 2004) (Table 1). We simulated the forest growth for time (*t*) = 300 years until the virtual forest attained the (stable) biomass of 375 *t*·ha^−1^ of a healthy natural mangrove forest (Putz and Chan 1986). This standing biomass was unbiased by the initial configuration and was used as a proxy for a “healthy forest” in further analyses. To minimize edge effects in modeling, this one-hectare patch of virtual forest is surrounded by a band of 10 m on all sides. Total simulated area was 120 × 120 m^2^, and sample area was 100 × 100 m^2^.

Each “impact” scenario started after the initial phase of 300 years when the biomass equilibrium was reached for the first time. Depending on the spatial scale of impact obtained from the Delphi technique (Fig. 2), a corresponding area in the virtual forest was cleared of trees (single killing event). For instance, if the spatial scale of the impact was 80%, then trees in 80% of the one-hectare virtual forest were killed. Furthermore, the regrowth (recruitment rate and growth of the trees within the impacted zone) was reduced (Table 1) based on the spatial scale and intensity of the impact obtained from the Delphi technique (Fig. 2). Though, in reality, not all impacts are reflected in terms of mortality, reduced recruitment or reduced regrowth, the current constraints of the model limit the modification to only these parameters.

After biomass reduction due to the impact, the subsequent biomass recovery of the forest was measured. The virtual forest was allowed to grow back to the predisturbance biomass, and the time required to attain the stable biomass was noted for each impact. The simulation run was halted after the initial biomass of 300–400 *t*·ha^−1^ was reached. For the control forest with no impact, a correction factor was included to account for the natural death in old forests. Each simulation scenario was repeated 100 times, and the median time frame was calculated for each impact.

## Results

### Mangrove Delphi

**Figure 3:**
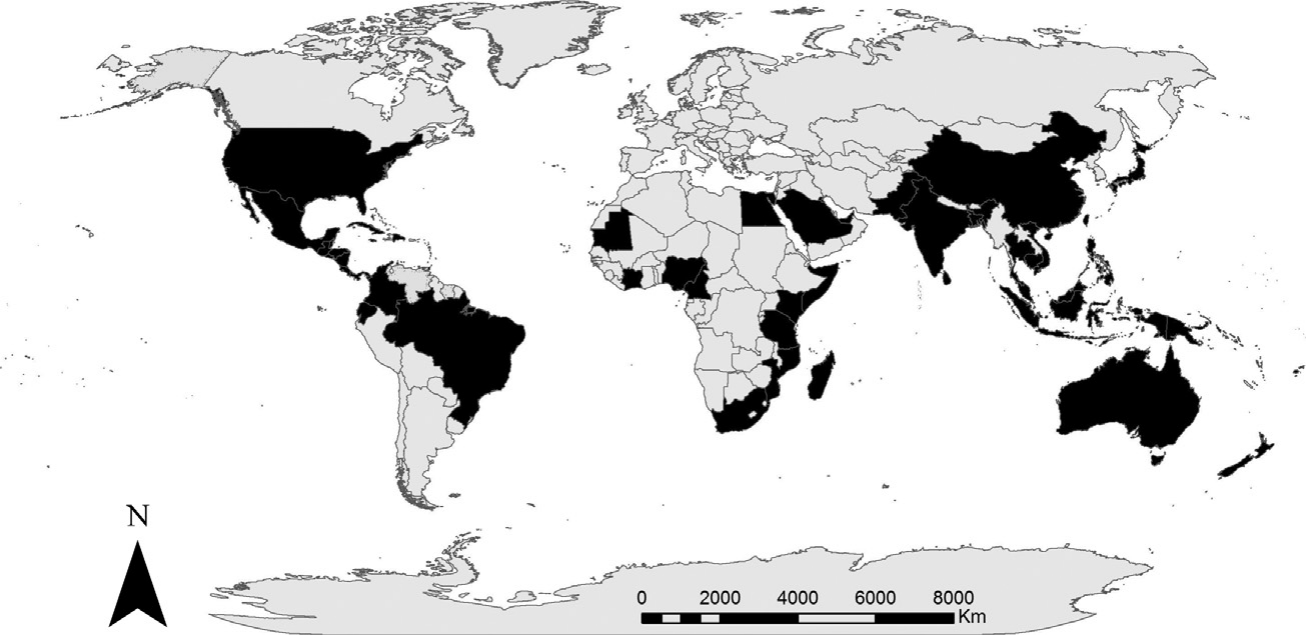
Map representing countries (colored black) where the experts have conducted primary research.

Thirty-five experts participated in the first round of the Delphi technique (34% of those invited), while nineteen experts participated in the second round (54% of the first-round participants). Figure 3 shows the 55 countries where respondents of the first round (*n* = 35) had carried out field research on mangroves. As of September 2013, the respondents had published 691 reviewed publications on mangroves, had been cited over 10,829 times (without self-citations), and had a median of 20 years of experience in mangroves (Table S1). Their cumulative expertise covers all mangrove species and environments (see Spalding et al. 2010).

### Constituents of the ecosystem

#### Consensus definition

The consensus definition that experts arrived at is as follows:

“Mangroves are woody plants that grow normally in tropical and subtropical latitudes along the land–sea interface, bays, estuaries, lagoons, and backwaters. These plants and their associated organisms constitute the “mangrove forest community” or “mangal”. The mangal and its associated abiotic factors constitute the “mangrove ecosystem”.” A complete list of all the definitions is given in Appendix S1.

**Figure 4:**
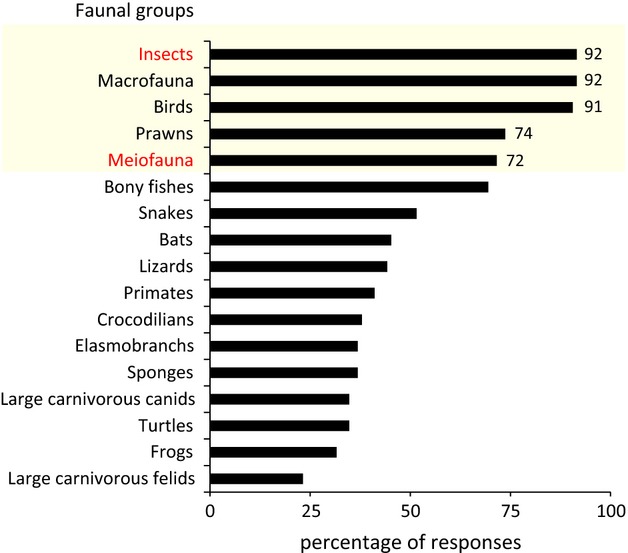
Ranking of the commonly observed faunal groups in a mangrove. The top five groups have been highlighted. The faunal groups indicated in red are those for which currently data are lacking.

The consensus list of mangrove species is shown in Table 2. Aboveground insects, macrofauna, birds, and meiofauna (metazoans passing though 1-mm sieve but retained by 38-*μ*m sieve) were reported to be the common faunal groups (>70% response). Insects were the most commonly observed faunal group. The ranking of the observations is shown in Fig. 4.

**Table 2:** Consensus list of mangrove plant species based on the first round of the Delphi technique. A threshold of 50% was chosen as the consensus. Nineteen experts answered this question. The taxonomy is based on Duke (1992) and Spalding et al. (2010), and conforms to the Angiosperm Phylogeny Group (APG III) classification system Stevens (2001)

Number	Name of the plant species	Percentage of responses
1	*Avicennia alba*	100
2	*Avicennia marina*	100
3	*Bruguiera gymnorrhiza*	100
4	*Bruguiera parviflora*	100
5	*Bruguiera sexangula*	100
6	*Ceriops decandra*	100
7	*Ceriops tagal*	100
8	*Rhizophora apiculata*	100
9	*Rhizophora mucronata*	100
10	*Rhizophora stylosa*	100
11	*Sonneratia alba*	100
12	*Sonneratia apetala*	100
13	*Sonneratia caseolaris*	100
14	*Sonneratia griffithii*	100
15	*Xylocarpus granatum*	100
16	*Xylocarpus moluccensis*	100
17	*Avicennia bicolor*	92
18	*Avicennia germinans*	92
19	*Avicennia officinalis*	92
20	*Bruguiera cylindrica*	92
21	*Bruguiera hainesii*	92
22	*Ceriops australis*	92
23	*Rhizophora mangle*	92
24	*Rhizophora racemosa*	92
25	*Rhizophora samoensis*	92
26	*Sonneratia ovata*	92
27	*Xylocarpus mekongensis*	92
28	*Avicennia integra*	85
29	*Avicennia schaueriana*	85
30	*Bruguiera exaristata*	85
31	*Heritiera littoralis*	85
32	*Kandelia candel*	85
33	*Lumnitzera littorea*	85
34	*Sonneratia lanceolata*	85
35	*Lumnitzera racemosa*	80
36	*Avicennia lanata*	77
37	*Avicennia rumphiana*	77
38	*Ceriops zipelliana*	77
39	*Excoecaria agallocha*	77
40	*Excoecaria indica*	77
41	*Heritiera fomes*	77
42	*Aegialitis rotundifolia*	69
43	*Aegiceras corniculatum*	69
44	*Aegiceras floridum*	69
45	*Heritiera globosa*	69
46	*Kandelia obovata*	69
47	*Laguncularia racemosa*	69
48	*Lumnitzera rosea*	69
49	*Aegialitis annulata*	62
50	*Nypa fruticans*	54
51	*Pelliciera rhizophoreae*	54
52	*Pemphis acidula*	54

**Figure 5:**
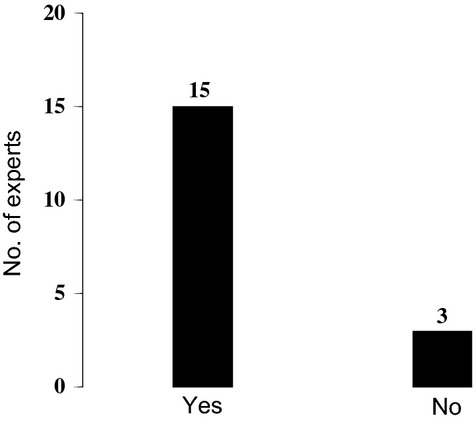
Responses to whether a mangrove forest (natural or planted) consisting of only one of the “true mangrove” tree species (mono-species stand) could be considered as a mangrove “ecosystem”. Majority of experts consider that a single mangrove (plant) species could be capable of forming a mangrove ecosystem.

A majority of respondents (83%) reported that a mono-specific stand could be considered a mangrove ecosystem (Fig. 5). The three respondents, who did not give a positive response directly, also considered a “mono-specific” forest to be a mangrove ecosystem provided certain conditions were met. These conditions are “tidal dynamics”, “dispersal”, and “adequate ecological interactions between the flora and fauna”.

### Ecological functions

**Figure 6:**
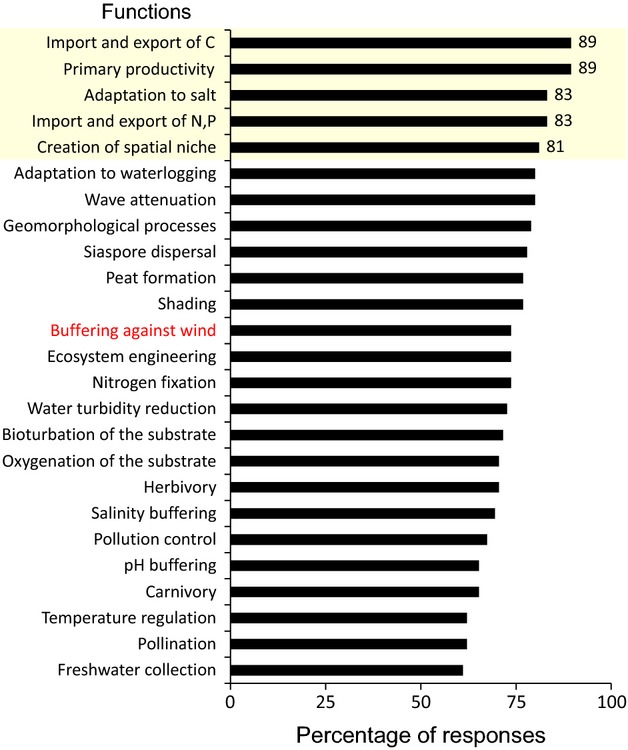
Ranking of the important ecological functions in a mangrove. The top five functions have been highlighted. Buffering against wind (indicated in red) was suggested by an expert in the first round, yet there were no published studies on this function at the time the survey was conducted.

The final ranking of the ecological functions is shown in Fig. 6. Import and export of carbon, primary productivity, adaptation to salinity, import and export of nutrients (e.g., N, P), and creation of spatial niche were all ranked highly (>80% response). The wind attenuation function in a mangrove was proposed by one expert in the first round of the survey and was ranked highly in the second round by the respondents (*n* = 7 respondents ranked it as “very important”).

### Maintenance of constituents and functions

#### Spatial scale and intensity

The ranking of the spatial scale and intensity of ten impacts are shown in Fig. 2. Degradation caused due to development (e.g., building of harbors and roads) was perceived to be the dominant impact across all countries. Tourism and degradation due to household uses (e.g., timber, fuelwood) were ranked second and third, respectively. There was an equal share of expertise in both highly developed countries and less developed countries (Table 3).

**Table 3:** List of countries where the experts had carried out the largest share of their fieldwork and for which they consequently answered the questions about maintenance of constituents and functions. The countries have been classified into highly developed (HD) and less developed (LD) based on a combination of categories “very high” and “high” for (HD) and low and medium for (LD), respectively, from the Human Development Index 2011(UNDP 2013). There was an almost equal share of respondents from both the (HD) and (LD) countries (*n* = 9 and 10, respectively)

Country name	Level of development	No. of responses
Australia	HD	4
Brazil	HD	2
Mexico	HD	2
USA	HD	1
Total (HD)		9
India	LD	3
South Africa	LD	2
Kenya	LD	1
Kiribati	LD	1
Indonesia	LD	1
Sri Lanka	LD	1
Bangladesh	LD	1
Total (LD)		10

#### Time frame for recovery

Strikingly, expert responses were equally split over the possibility of natural restoration of functionality (*n* = 7 for both positive and negative responses). The majority of experts suggested that mangroves could regain functionality in the degraded areas with human assistance (*n* = 13 of 14). Only one expert perceived the mangroves in his/her study area were too degraded for functional restoration, even with human assistance.

**Figure 7:**
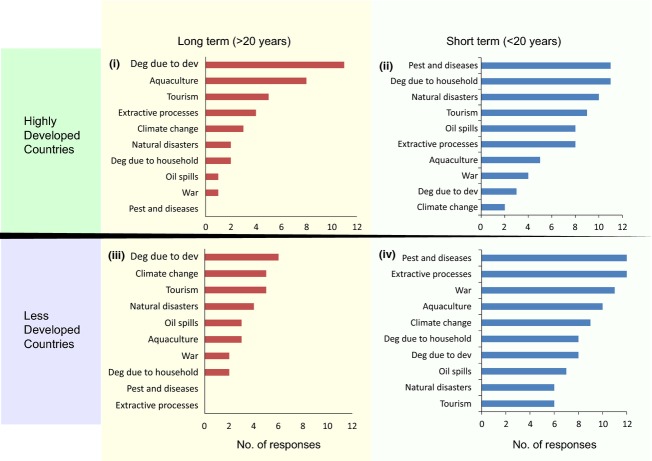
Time frames for restoration of functionality based on the Delphi technique are shown in four sections. The responses were divided into “highly developed” (HD) and “less developed” (LD) based on the classification of the countries selected by the experts using the Human Development Index (UNDP 2013). “Short term” (ST) refers to the restoration process to be less than 20 years while “long term” (LT) refers to the restoration process taking longer than 20 years. The four combinations are as follows: (i) HD-LT, (ii) HD-ST, (iii) LD-LT, and (iv) LD-ST. The *x*-axis denotes the total number of responses for both natural- and human-mediated restoration processes for each of the four categories. Restoration after the impact “degradation due to development” was estimated to be a long-term process both in HD and in LD countries. Deg, degradation; dev, development.

Restoration of functionality after development related degradation is considered to be a long-term process (ranked highest) in all scenarios (highly developed and less developed countries) (Fig. 7i and iii). In highly developed countries, the second-, third-, and fourth-ranked impacts were aquaculture, tourism, and extractive processes (e.g., sand mining), respectively. In less developed countries, they were climate change, tourism, and natural disasters.

Restoration of functionality after pest and disease attacks was considered to be a short-term process in both highly developed and less developed countries (Fig. 7ii and iv). Respondents who have worked in highly developed countries considered restoration after degradation for household uses, natural disasters, and tourism to be short-term processes. On the other hand, respondents who had worked in less developed countries ranked restoration after extractive processes, aquaculture, and war higher as short-term processes.

#### Prediction of recovery time based on modeling

**Figure 8:**
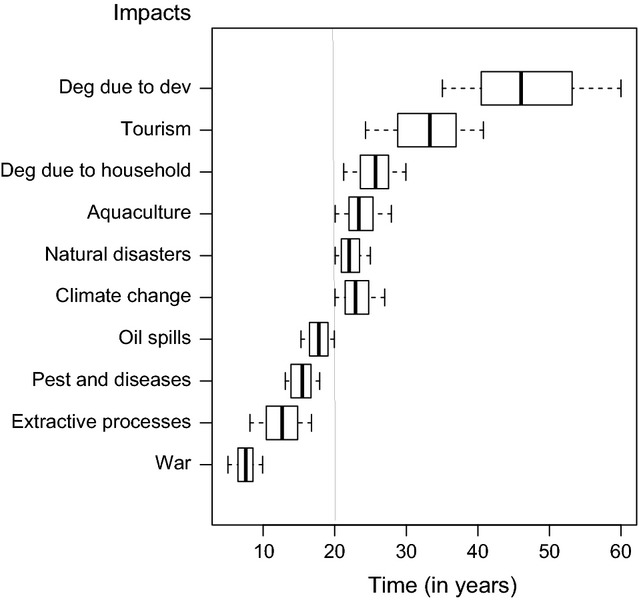
Boxplot of time required to achieve a biomass asymptote of 375t/ha in *Rhizophora apiculata* after impact. Both spatial scale and intensity of impacts (data from the Delphi technique) were simultaneously incorporated into the simulation. Each box represents the results of 100 simulations. Restoration after the impact “degradation due to development” was estimated to require more than 40 years.

The results obtained by the simulation runs in the KiWi model indicate that the time required for returning to an asymptotic biomass of 375 *t*·ha^−1^ was greater than 20 years for six of the impacts: development, tourism, household uses, aquaculture, natural disasters, and climate change (Fig. 8). The longest time for functional recovery was noted for the impact “degradation due to development” (greater than 40 years) (Fig. 8).

## Discussion

Despite the variation within mangrove ecosystems in form, structure, and function, the experts reached a consensus on a large range of issues even though they might have worked in geographically disparate mangrove areas. Moreover, the breadth and validity of knowledge that could be accessed by the Delphi technique was ensured by addressing all traceable experts on mangroves meeting the requirements given in Appendix S1. The number of respondents in this Delphi survey was also high, given that some published surveys using the Delphi technique had three to five experts (Crabbe et al. 2010; Ochoa-Gaona et al. 2010) with the usual number of respondents being 15–35 (Donohoe and Needham 2009). In addition, the experts who participated in the Delphi technique had a median of 20 years of experience dealing with mangroves. In an ideal scenario, conservation decisions should be guided by empirical data may be needed. However, given the urgency driven by rapid mangrove forest loss and the complexity of conservation decisions, participatory approaches (involving respondents with relevant expertise), such as that used in this study, are crucial. (O'Faircheallaigh 2010).

The results of this study have implications for three aspects relevant to mangrove ecosystems: (1) ecological research, (2) conservation and management of mangrove forests, and (3) policy.

### Ecological research

The consensus definition and list of mangrove species can be used as a benchmark for further research on vegetation in mangroves. This list could also be used for mapping the ecological linkages between mangrove plant species and other biota in the mangrove ecosystem.

The function of wind attenuation by mangroves was proposed by one of the respondents in the first round of the Delphi technique and subsequently ranked highly in the second round. This ecological function needs further investigation as there is very little empirical evidence in the published literature on this aspect (Badola and Hussain 2005) although wave attenuation has been studied extensively (Quartel et al. 2007; Mei et al. 2011). This highlights the strength of the Delphi technique in facilitating the emergence of new insights that could not have been obtained through a review or rigorous meta-analysis of quantitative data.

In the ecological context of mangroves, the experts reached a consensus that a mono-specific forest, whether natural or planted, is potentially able to constitute a mangrove ecosystem (if we also include the three guarded responses). This is contrary to the existing paradigm where high species diversity is considered important for the ecological “insurance” for ecosystem functions and the subsequent resilience of an ecosystem (Naeem et al. 2012). Prior to this research, mono-specific mangrove plantations were being critiqued by restoration ecologists as it was feared that they might not be as ‘functional’ as mixed species stands Ellison 2000, Lewis 2005, Walters 2000). The fundamental message in all these earlier works was that in order to have a diverse set of mangrove ecosystem functions we need to plant multiple species. This apprehension was fuelled by research in temperate forest ecosystems which normally have much higher levels of plant diversity than mangrove ecosystems and where monocultures were raised primarily for timber (Perry 1998). However, in harsh environmental conditions with tremendous physiological limitations for trees to grow, mangrove plants have evolved toward nonredundant systems. Perhaps the results indicate that even though plant species diversity and richness are low in this ecosystem, mangrove plants are highly “functional”, as they can fulfill a multitude of ecological functions for which many plant species may be required in other ecosystems. This aspect calls for further research attention on the functionality of mangrove plant species. Key functions in a mangrove e.g. shading (uncommon on a coast), carbon input, water turbidity reduction etc. can be accomplished by a mature forest stand even it is tree species poor. This clearly sets mangroves apart from the rest of forested ecosystems. This is a big shift in our perception about the diversity-functioning relationship and thereby restoration for this ecosystem.

The experts reported that birds, insects, macrofauna, and meiofauna were commonly observed in mangroves. Participant bias was not observed here, as respondents were experts in various domains ranging from socioeconomics to individual-based modeling (Table S1). This study draws attention to the paucity of information specifically for insects and meiofauna in mangroves as their macroecological patterns still remain to be investigated (Dye 1983; Sasekumar 1994; Alongi et al. 1998). As mangrove ecosystems are situated in a zone where all three realms (marine, freshwater, and terrestrial) may occur adjacently, the nature of faunal groups in mangroves is ecologically and evolutionarily interesting. The theory of niche conservatism (Pearman et al. 2008; Petitpierre et al. 2012) predicts that evolutionary transitions among these three realms are relatively rare within the major clades of plants, animals, fungi and microbes, and that many of the clades are predominantly found in only one or two of the three realms (Grosberg et al. 2012).

### Management and conservation of mangroves

The mangrove species list shown in Table2 can be used as a baseline to identify locally available species for future mangrove restoration programmes. This list can also be used to delimit mangrove area or quantify mangrove area loss/gain over time, based on the mangrove species naturally occurring in that specific location. This is particularly relevant for avoiding ambiguity with “mangrove associate species” sensu Tomlinson (1986) and exotic species while calculating mangrove forest area or restoration status.

The Delphi analysis points to the fact that mangrove ecosystems are unique in their ecological functionality. Restoration measures should thereby focus on the regeneration of a multitude of ecological functions. Given that at a regional scale, 40% of mangrove species on/along the Pacific and Atlantic coasts of Central America are threatened with extinction, and globally 16% of mangrove species are at an increased threat of global extinction (Polidoro et al. 2010), it is imperative to protect these highly productive ecosystems, even though their plant species richness is low. Even the remaining 84% of species considered “not threatened” at a global level by the International Union for the Conservation of Nature and natural resources (Polidoro et al. 2010) may be severely threatened at the local level. In fact, the low vascular species diversity of mangroves combined with the wide biogeographic distribution of mangroves makes it impossible to ever rank a mangrove forest as a “hotspot” based on the criteria (0.5% endemic with 70% loss of vegetation) set by Myers et al. (2000).

The ranking of the ecological functions provides a baseline for ecological restoration of mangroves (Fig 6). The top five functions ranked by the experts in our study are influenced by hydrology. However, this aspect has been routinely neglected in mangrove management, resulting in dire consequences (Dahdouh-Guebas et al. 2005a,b). This is despite the fact that past research has repeatedly stressed the importance of hydrology in mangrove ecosystems (Lewis and Gilmore 2007; Bosire et al. 2008). Future mangrove management should therefore pay careful attention to hydrologic connectivity in mangroves.

The results from the Delphi technique and modeling exercise indicate that degradation of mangroves due to development has a major impact on mangroves across all countries and would be the hardest to restore. This is in contrast to published literature where aquaculture is reported as the major driver of mangrove loss (Valiela et al. 2001; Barbier and Cox 2003; Bird et al. 2004; Primavera 2006; Pattanaik and Narendra Prasad 2011). Valiela et al. (2001) report that 38% of global mangrove loss is due to shrimp culture and further 14% is due to fish culture. Barbier and Cox (2003) did an analysis of 89 countries using an economic model and concluded that the level of economic development was positively linked to greater mangrove area. They also concluded that in low- and middle-income countries, economic growth did not affect mangrove area. Clearly, the scenario presented in this paper sheds a different light. Based on the consensus of experts who have worked in mangroves for over 20 years, we found that development in coastal settings was the major factor driving mangrove loss.

Restoration of functionality after tourism-related damage is considered to be a long-term process in both highly developed and in less developed countries. Rapid increase in tourism in the Caribbean Islands and in West Africa has already been documented by Avau et al. (2011) and Satyanarayana et al. (2012). Future conservation and management plans in mangrove areas should take the impact of tourism-related activities on the ecosystem functioning of mangroves into account, in addition to the design of locally adapted management actions.

### Policy recommendations

We draw inspiration from the European Union's Habitat Directive 92/43/EEC on the “favorable conservation status” of a natural habitat as an example of an international conservation policy. In light of the results of our study, we recommend the establishment of newly designed conservation and restoration programmes that move well beyond the planting of mangroves. Conservation must be viewed from the functionality perspective at all scales unlike current restoration programmes that are largely oriented toward the highest probability of establishment success of mangrove seedlings. The latter strategy does not necessarily guarantee the restoration of ecosystem functions. If however, planting is the only option, we recommend the consultation of the species list (for locally growing mangroves) presented here to better consider the multitude of functions provided by diverse mangrove species. Moreover, while much mangrove research and concerns expressed in the literature relate to overexploitation and land use conflicts at a small scale, our study clearly shows that more attention must be focused on avoiding and mitigating mangrove loss at larger scales by coastal development (such as building of roads, ports and harbors, etc.). In addition, we recommend that restoration and conservation programmes should be linked to local livelihood requirements. This is crucial particularly in developing countries where a considerable portion of the population is very dependent on mangroves (Walters et al. 2008; Nfotabong et al. 2009; Liquete et al. 2013; Uddin et al. 2013).

### Limitations

The present study draws attention to the potential of combining different techniques (Delphi technique with modeling) to provide valuable support and validation for restoration programmes. Models like KiWi can be used to forecast the development of restored forests in terms of forest structure and biomass. Both forest structure and biomass are related to some important ecosystem functions, and the model outputs can thus be used as a first indication for restoration, but not more. To the best of our knowledge, there is still a dearth of models that quantifying the actual impacts of climate change, pest infestation, or natural hazards on mangrove forest dynamics. For this new mangrove forest, models are required, and these must explicitly address the responses of mangrove trees to such environmental factors. In addition, the responses to the impacts “climate change” and “war” should be considered with caution, because the simulations were based on the inputs from the Delphi technique where (1) some experts were not comfortable with “climate change” being an impact or (2) there was lack of experts from war-affected countries.

The application of the Delphi technique has demonstrated that the compilation of expert opinions is a valuable tool for resolving complex issues. However, it is certainly not error-free. Defining and choosing an expert is a subjective process (even though a priori criteria may be applied) and has been one of the foremost challenges in using the Delphi technique (Hasson et al. 2000). Consequently, we have attempted to be as inclusive as possible and have clearly stated the criteria used to choose respondents for this survey (see supplementary section Appendix S1).

There may also be inherent biases and subjectivity in some of the responses for instance in estimating the spatial scale and extent of the ten different impacts. Similarly, the responses of the commonly observed fauna could also be biased by the fact that most of the observations may have been made at low tide, when mangroves are usually accessed and are mostly from scientific experts rather than local communities.

## Conclusion

In this study, we addressed three aspects of the ecological functionality of mangrove ecosystems from a global perspective and using an approach based on expert knowledge and modeling. The consensus list of mangrove species generated in this study can be used to identify locally occurring mangrove species and as a tool in both future ecological research and mangrove management. Our study also identifies gaps in the existing literature on the ecosystem functioning of mangroves (*viz*. wind attenuation function that was mentioned by only one respondent in the first round and subsequently taken up by many respondents in the second round). Our results indicate that degradation of mangroves due to development has a major impact on mangroves across all countries and would take the longest time to restore. This is contrary to the notion widely prevalent in existing literature where much attention has been paid to local overexploitation as well as to aquaculture for mangrove loss. Moreover, our study also highlights the strengths of using a combination of techniques (here the Delphi technique and modeling) to derive new insights into controversial aspects of mangrove ecology and thus forms a baseline for future investigations.

## Supporting Information

**Table S1.** Detailed information about the 35 experts who contributed information for this Delphi study. Included in the table are their number of publications largely on mangroves, years of experience, research focus, personal webpages, and total number of citations (without self-citations) for their mangrove articles.

**Appendix S1**. (a) Detailed methodology of the Delphi survey in this study. (b) List of all the definitions for the mangrove ecosystem.
