# Supplementary material for: Using expert knowledge and modeling to define mangrove composition, functioning, and threats and estimate time frame for recovery
Source: Ecol Evol. 2014 May 8;4(11):2247–62. doi: 10.1002/ece3.1085 (PMC4201438; doi:10.1002/ece3.1085)
Supplement: Supplementary file 2 — Appendix S1. (a) Detailed methodology of the Delphi survey in this study. (b) List of all the definitions for the mangrove ecosystem. [file ece30004-2247-sd2.docx]

**Supplementary Material for**

Using expert knowledge and modelling to define mangrove composition, functioning and threats and estimate time-frame for recovery

Authors: Nibedita Mukherjee^1,2*^, William J. Sutherland^3^, Md. Nabiul Islam Khan^1^, Uta Berger^4^, Nele Schmitz^5^, Farid Dahdouh-Guebas^1,2,‡^ and Nico Koedam^2,‡^

*^1^Laboratory of Systems Ecology and Resource Management, Université Libre de Bruxelles, CP 169, Avenue F.D. Roosevelt 50, B-1050 Brussels, Belgium, ^2^Laboratory of Plant Biology and Nature Management, Vrije Universiteit Brussel, Pleinlaan 2, B-1050 Brussels, Belgium, ^3^Conservation Science Group, Department of Zoology, University of Cambridge, , Cambridge CB2 3EJ, England; ^4^Institute of Forest Growth and Forest Computer Sciences, TU Dresden, P.O. 1117, 01735 Tharandt, Germany, ^5^Institute of Botany, BOKU Vienna, Gregor Mendel Str. 33, 1180-Vienna, Austria*

*Correspondence: Nibedita Mukherjee, Laboratory of Systems Ecology and Resource Management, Université Libre de Bruxelles, CP 169, Avenue F.D. Roosevelt 50, B-1050 Brussels, Belgium. E-mail: nibedita.41282@gmail.com

‡Co-last author

**This file contains:**

a: Detailed methodology of the Delphi survey in this study.

b: List of all the definitions for the mangrove ecosystem.

**a) DELPHI SURVEY**

*Selection of experts*: The experts were chosen based four criteria

1. Number of peer-reviewed publications related to mangrove ecology: We searched for the keywords ‘mangrove’ and ‘ecology’ appearing together in ISI Web of Science® and identified the first 250 authors irrespective of co-authorship or author position. This list of 250 authors was then manually sorted.
2. Publications in high impact factor journals: We identified authors based on key research articles on mangroves in three high impact factor journals (Impact Factor>ten) - Science, Annual Review of Marine Science and Public Library of Science.
3. CREC project: We also selected mangrove experts working on an international European-Commission funded collaborative project on mangroves termed as Coastal Research network on Environmental Changes (2010-2014) in five continents (http://www.forst.tu-dresden.de/CREC/).
4. Managers and restoration biologists: We also invited mangrove managers and on-ground restoration biologists who were/are involved in mangrove research.

Based on the above short listing a total of 106 mangrove experts were finally selected. Care was taken to select experts outside our research group and the authors who supervised this research did not participate in this survey even though both FDG and NK are mangrove experts.

*Number of questions asked*: In the first round, eleven questions were asked. In the second round, the questionnaire was slightly modified based on the comments received in the first round and eighteen questions were asked.

*Survey design and software*: The questionnaires in both rounds consisted of a mixture of open ended and close ended questions. Not all questions were compulsory. In some questions experts were asked to rank options on a Likert scale of 1-5 ([Likert, 1932](#_ENREF_6)). The entire survey was conducted online and a website was designed specifically for this survey ([Mukherjee, 2011](#_ENREF_7)). The online survey questionnaires in both rounds were designed using Google Forms.

*Procedure of the survey*: The Delphi survey consisted of 5 steps. (i) We prepared the first round of questions of the Delphi survey and sent an online invitation for participation to the selected 106 experts. (ii) The respondents completed the survey and sent it back to us. (iii) We analysed the responses and prepared a feedback report which was uploaded on the website. (iv) The second round of questions was prepared based on the responses in the first round. The experts who participated in the first round were requested to participate in the second round. Thus the participants were self-selected in the second round. (v) Thereafter, the second round responses were analysed, compiled into a feedback report and uploaded on the website. The Delphi survey was terminated after the second round.

The survey was anonymous while it was being conducted, based on the principle of the classical Delphi method. The respondents were given the choice of being acknowledged or remaining anonymous at the end of the second round of the survey. Names of those mangrove experts who wished to be acknowledged can be found in the Table S1.

*Analysis*: We used MS Excel for the exploratory data analysis of the ordinal data. The statistical package ‘R’ was used for modelling and creating graphs.

**b) DEFINITIONS FOR THE MANGROVE ECOSYSTEM**

**Definitions from the literature**

| **No.** | **Definition** | **Authors** | **Source type** |
| --- | --- | --- | --- |
| 1 | Mangroves in the more limited sense may thus be defined as tropical trees restricted to intertidal and adjacent communities. Another definition of mangal is a community that contains mangrove plants. | [Tomlinson (1986](#_ENREF_9)) | Book |
| 2 | Mangroves are woody plants that grow normally in tropical and sub-tropical latitudes along the land-sea interface, bays, estuaries, lagoons, backwaters, and in the rivers, reaching upstream up to the point where the water still remains saline (Quasim, 1998). | [Kathiresan and Qasim (2005](#_ENREF_5)) | Book |
| 3 | Trees, shrubs, palms, or ground ferns growing in the zone between high and low tide marks. | [Field *et al.* (1996](#_ENREF_4)) | Book |
| 4 | A mangrove is a tree, shrub, palm or ground fern, generally exceeding one half metre in height, that normally grows above mean sea level in the intertidal zone of marine coastal environents and estuarine margins. A mangrove is also the tidal habitat comprising such trees and shrubs. | [Duke (2006](#_ENREF_2)) | Book |
| 5 | Mangroves are trees or large shrubs, including ferns and a palm, which normally grow in or adjacent to the intertidal zone and which have developed special adaptations in order to survive in this environment. The term mangrove refers both to these trees and to the communities they form, which range from narrow fringes of stunted trees tracing desert margins, to broad forests fragmented into myriad patches by the braiding channels of a delta. | [Spalding *et al.* (2010](#_ENREF_8)) | Book |
| 6 | Mangrove ecosystems are coastal wetlands dominated by woody plants that span gradients in latitude (30°N to 30°S), tidal height (<1m to >4m), geomorphology (oceanic islands to riverine systems), sedimentary environment (peat to alluvial), climate (warm temperate to both arid and wet tropics), and nutrient availability (oligotropic to eutropic). | [Feller *et al.* (2010](#_ENREF_3)) | Paper |
| 7 | "Mangrove" is an ecological term referring to a taxonomically diverse association of woody trees and shrubs that form the dominant vegetation in tidal, saline wetlands along tropical and subtropical coasts. | [Ball (1998](#_ENREF_1)) | Paper |
| 8 | Mangroves refer to a unique group of forested wetlands that dominate 240X103 km2 of the intertidal zone of tropical and subtropical coastal landscapes from river deltas, lagoons and estuarine settins to islands in oceanic formations (non-continental). | [Twilley (2008](#_ENREF_10)) | Book |

**New definitions proposed by respondents in Round 1**.

9) Mangroves are woody plants that grow normally in tropical and subtropical latitudes along the land-sea interface, bays, estuaries, lagoons, and backwaters. These plants and their associated organisms constitute the 'mangrove forest community' or 'mangal'. The mangal and its associated abiotic factors constitute the "mangrove ecosystem".

10) Mangals in the more limited sense may thus be defined as tropical trees restricted to intertidal and adjacent communities whereas Mangrove is the coastal wetland ecosystem dominated by woody plants that span in inter tropical latitudes, tidal height (<1m to >4m), geomorphology (oceanic islands to riverine systems), sedimentary environment (peat to alluvial), climate (warm temperate to both arid and wet tropics), and nutrient availability (oligotrophic to eutrophic).

11) Intertropical salt tolerant communities subject to flooding at the continental and sea-water interface. Mangroves are characterized by the presence of woody plants that display anatomical and physiological features such as vivipary and root modifications to withstand substrate anaerobiosis.

12) #5 above plus: The ecosystem includes communities of mangroves, and in some locales associated salt marsh, marine fauna and terrestrial (avian, insect, etc.) fauna.

13) Mangroves are woody plants that grow normally in tropical and sub-tropical latitudes along the land-sea interface, bays, estuaries, lagoons, backwaters, and in the rivers, reaching upstream up to the point where the water still remains saline. The term mangrove refers both to these trees and to the communities they form.

14) Mangroves are inter-connecting; saline wetlands that include but are not restricted to trees or shrubs found within the intertidal zone, but include mudflats and salt flats. “Mangrove” refers to the whole interactive tidal zone, including communities they form and support.

**References**

Ball, M.C. (1998) Ecophysiology of mangroves. *Trees*, **2**, 129-142.

Duke, N.C. (2006) *Australia's Mangroves: The Authoritative Guide to Australia's Mangrove Plants*. University of Queensland.

Feller, I.C., Lovelock, C.E., Berger, U., McKee, K.L., Joye, S.B. & Ball, M.C. (2010) Biocomplexity in Mangrove Ecosystems. *Annual Review of Marine Science*, **2**, 395-417.

Field, C.D., Esosystems, I.S.f.M. & Organization, I.T.T. (1996) *Restoration of Mangrove Ecosystems*. International Society for Mangrove Ecosystems.

Kathiresan, K. & Qasim, S.Z. (2005) *Biodiversity of Mangrove Ecosystems*. Hindustan Publishing Corporation, New Delhi.

Likert, R. (1932) A Technique for the Measurement of Attitudes. *Archives of Psychology*, **140**, 1-55.

Mukherjee, N. (2011) *Welcome to the Delphi Survey on Mangroves*. Available at: https://sites.google.com/site/trialmangrovesblank/home (accessed

Spalding, M., Kainuma, M. & Collins, L. (2010) World atlas of mangroves. In. Washington, DC : Earthscan,, London, UK ;.

Tomlinson, P.B. (1986) *The botany of mangroves*. Cambridge University Press, Cambridge.

Twilley, R.R. (2008) Mangrove Wetlands. *Encyclopedia of Ecology* (ed. by S.E. Jørgensen and B.D. Fath), pp. 2198-2208. Academic Press, Oxford.
